# Diacylglyceryl-*N*,*N*,*N*-trimethylhomoserine-dependent lipid remodeling in a green alga, *Chlorella kessleri*

**DOI:** 10.1038/s42003-021-02927-z

**Published:** 2022-01-11

**Authors:** Yutaro Oishi, Rie Otaki, Yukari Iijima, Eri Kumagai, Motohide Aoki, Mikio Tsuzuki, Shoko Fujiwara, Norihiro Sato

**Affiliations:** School of Life Sciences, Tokyo University of Pharmacy and Life Sciences, Horinouchi 1432-1, Hachioji, Tokyo, 192-0392 Japan

**Keywords:** Abiotic, Water microbiology

## Abstract

Membrane lipid remodeling contributes to the environmental acclimation of plants. In the green lineage, a betaine lipid, diacylglyceryl-*N*,*N*,*N*-trimethylhomoserine (DGTS), is included exclusively among green algae and nonflowering plants. Here, we show that the green alga *Chlorella kessleri* synthesizes DGTS under phosphorus-deficient conditions through the eukaryotic pathway via the ER. Simultaneously, phosphatidylcholine and phosphatidylethanolamine, which are similar to DGTS in their zwitterionic properties, are almost completely degraded to release 18.1% cellular phosphorus, and to provide diacylglycerol moieties for a part of DGTS synthesis. This lipid remodeling system that substitutes DGTS for extrachloroplast phospholipids to lower the P-quota operates through the expression induction of the *BTA1* gene. Investigation of this lipid remodeling system is necessary in a wide range of lower green plants for a comprehensive understanding of their phosphorus deficiency acclimation strategies.

## Abstract (web summary)

Oishi et al. report that the synthesis of a betaine lipid, diacylglyceryl-*N,N,N-trimethylhomoserine* (DGTS) in a green alga Chlorella kessleri replaces phosphatidylcholine and phosphatidylethanolamine, the two most abundant phospholipids, during phosphate deprivation. This study provides insights into the understanding of the phosphorus-deficiency acclimation strategies via lipid remodeling in lower green plants.

## Introduction

Polar lipids that form lipid bilayers are the foundation for the construction of membranes, and embedded membrane proteins contribute to membrane functionality. Over two decades, information has accumulated on the remodeling of membrane lipids crucial for acclimation to some environmental stresses in photosynthetic organisms or for compensation of their mutational loss of some polar lipids^1,2^. This lipid remodeling includes phosphorus (P)-limitation stress-induced replacement of one anionic lipid, phosphatidylglycerol (PG), with another anionic and non-P lipid, sulfoquinovosyl diacylglycerol (SQDG), in the membranes of plant chloroplasts and in those of some bacteria, including the postulated ancestor of chloroplasts, cyanobacteria. The replacement of PG by SQDG is thought to maintain the charge balance of photosynthetic membranes at a certain level for the proper performance of photosynthesis^2^. Meanwhile, in seed plants, in particular, in the roots and shoots, P-limitation stress also induces distinct lipid remodeling that substitutes another non-P lipid, digalactosyl diacylglycerol (DGDG), for phosphatidylcholine (PC) in extraplastid membranes^1^.

**Fig. 1:**
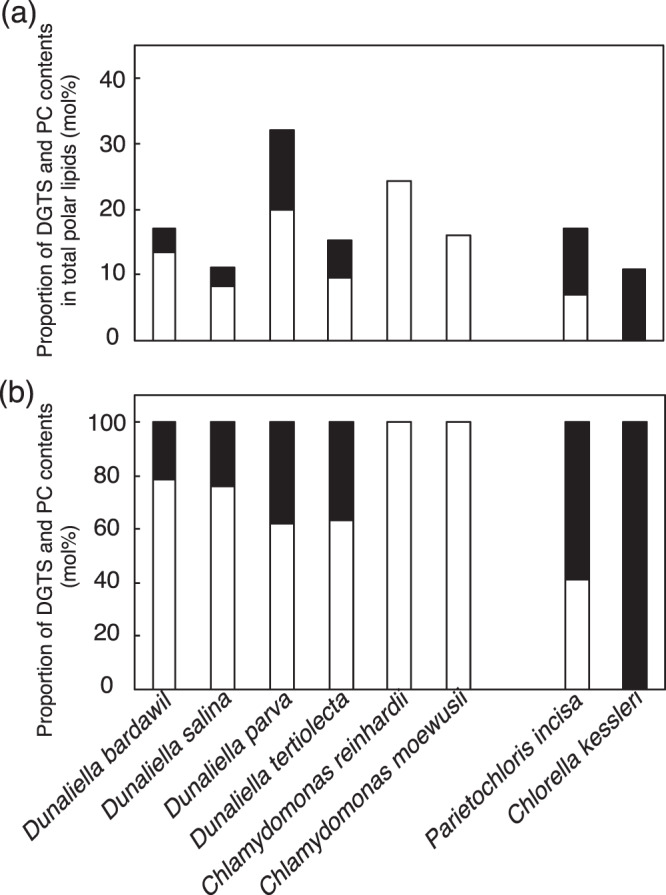
DGTS and PC contents in green algae grown under +P conditions. **a** Respective contents of DGTS and PC relative to that of total polar lipids. **b** The proportion of DGTS and PC contents. White and black bars indicate the contents of DGTS and PC, respectively. The values were obtained from previous reports^10–15, 17^.

Diacylglyceryl-*N*,*N*,*N*-trimethylhomoserine (DGTS), which is a membrane lipid, possesses both a positively charged trimethylammonium group and a negatively charged carboxyl group. This non-P betaine lipid is therefore categorized as a zwitterionic lipid^3^. DGTS is prevalently distributed in evolutionarily lower photosynthetic organisms in the green lineage, including green algae and nonflowering plants such as mosses and ferns^4–6^. Since no DGTS-containing seed plants have been found, it seems that DGTS plays some specific role in lower green plants. DGTS is similar to another zwitterionic lipid, PC, with respect to chemical and biophysical properties^7^. It is generally accepted that in lower green plants, PC is localized mainly in extraplastid membranes, as observed in seed plants^8^. Meanwhile, information on the subcellular localization of DGTS is restricted to some green algal species, including its occurrence in the plasma membranes of a green alga, *Dunariella. salina*^9,10^. In this context, it is of note that the DGTS content tends to be high in species that possess a low content of PC (Fig. 1a, b), which can be interpreted as reflecting functional substitution of DGTS for PC in extrachloroplast membranes^6^.

Concerning green algae, *Dunaliella* and *Chlamydomonas* species, which belong to Chlorophyceae, possess DGTS as one of the major lipids that represents as high as 8.4–24.3 mol% of total polar lipids, and the PC content is low (Fig. 1)^10–14^. In particular, *Chlamydomonas reinhardtii* and *Chlamydomonas moewusii* are extreme in that they exclusively possess DGTS with no PC. In contrast, DGTS is lower in quantity than PC in the green algal species Treboxiophyceae, as in *Parietochloris incisa* (Fig. 1)^15^. This tendency is more obvious in *Chlorella* species in particular: DGTS amounts to only 1.3% in *Chlorella fusca*, and more importantly, it is absent in *Chlorella kessleri* (*Parachlorella kessleri* previously known as *C. vulgaris*) and *Chlorella pyreniodosa* with high PC contents (Fig. 1)^6,16–18^.

DGTS is observed in species other than lower green plants, although within taxonomically narrow ranges. Several species including a secondary endosymbiotic alga of the red lineage, *Nannochloropsis oceanica*, an anoxygenic photosynthetic bacterium, *Rhodobacter sphaeroides*, and a fungus, *Flammulina velutipes*, were shown to remodel lipids by substituting DGTS for PC upon P-limitation stress^19–21^. Moreover, a DGTS-loss mutation impaired acclimating cell growth under P-limited conditions in *N. oceanica*^20^. To our knowledge, however, information on lipid remodeling is scarce for lower green plants under P-limited conditions, despite its potential involvement in their mechanism of acclimation to the P stress.

*C. kessleri* is industrially attractive because of its ability to synthesize triacylglycerol (TG) at high levels^22–24^. We previously reported that the membrane lipid metabolism in *C. kessleri* is similar to that in a seed plant, *Arabidopsis thaliana*, rather than to that in another green alga, *C. reinhardtii*. First, in *C. kessleri* as well as in *A. thaliana*, PC is present with DGTS being completely absent whereas in *C. reinhardtii*, DGTS contrarily exists in the absence of PC^25,26^. Second, lipid synthesis in chloroplast membranes depends on the cooperation of two lipid biosynthetic pathways, i.e., the prokaryotic pathway within chloroplasts and the eukaryotic pathway via the ER in *C. kessleri*, as in *A. thaliana*, whereas it proceeds predominantly through the prokaryotic pathway in *C. reinhardtii*^17,25–27^. Intriguingly, during our study on polar lipids of *C. kessleri* under stress conditions for the induction of TG accumulation, an unidentified lipid with a similar Rf value to that of DGTS appeared (Supplementary Fig. 1), which indicated the necessity of reevaluating membrane lipid metabolism in *C. kessleri*.

In this study, we investigated lipid remodeling for acclimation to P-starved conditions in *C. kessleri* in view of well-known quantitative increases in DGTS under such P-stress conditions, and regulatory expression patterns of the genes involved in this remodeling. The results were interpreted as showing that *C. kessleri* is endowed with a sophisticated regulatory mechanism that almost completely replaces extraplastid phospholipids including PC, with DGTS upon necessity, and that this lipid remodeling is responsible for Pi recycling.

## Results

### P-starvation induced cell growth defects in *C. kessleri*

**Fig. 2:**
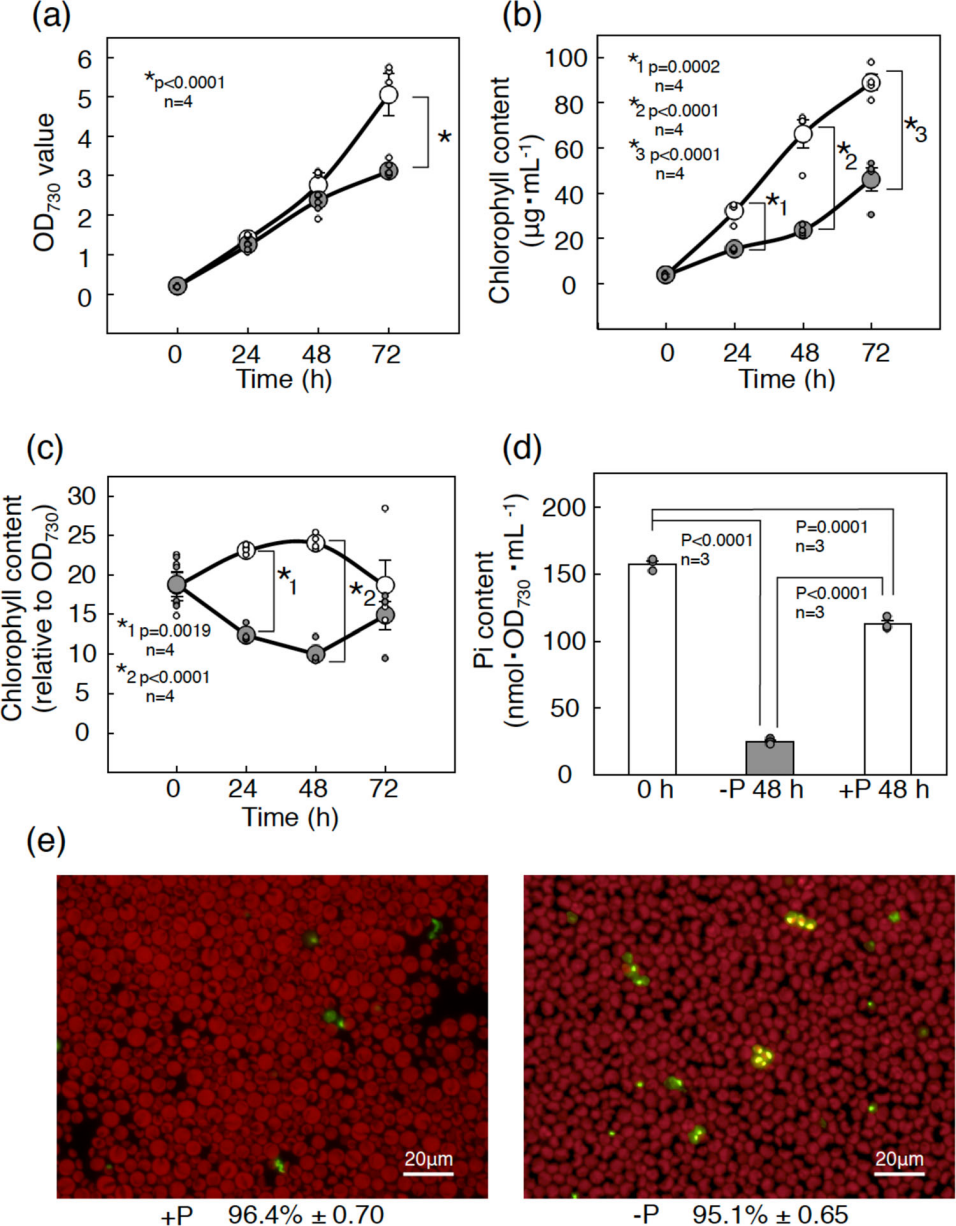
Effects of −P stress on physiological behavior in *C. kessleri* cells. **a** Cell growth on the basis of the OD_730_ value of the culture. **b** Chl content in the culture. **c** The cellular Chl content obtained through estimation of the relative ratios of the values in (**b**) to those in (**a**). White and gray circles indicate cells grown under +P and −P conditions, respectively. **d** Pi contents measured in cells grown under +P conditions (0 h), and in ones grown further for 48 h under +P and −P conditions. **e** Images of the cells emitting red auto-fluorescence of Chl and/or the green fluorescence of SYTOX bound to chromosomal DNA. The survival ratio of the cells was determined through estimation of the proportion of the number of viable cells emitting only red fluorescence, relative to that of total cells, including non-viable cells with emission of green fluorescence only and with that of both red and green fluorescence. The values shown are averages ± SEM for four biological replicates (**a**–**c**), and those for three biological replicates (**d**, **e**). Data were analyzed by one-way ANOVA with multiple comparison by Tukey–Kramer’s test. Some dots are overlapped (refer to source data in Supplementary Data 1).

This study investigated the polar lipid composition under conditions of P starvation (−P) in *C. kessleri* to reevaluate its lipid metabolism. We first examined the effects of −P on cell growth in *C. kessleri*. Compared with P-repletion (+P) conditions, −P conditions had little deleterious effect on cell growth for the first 24 h; however, they caused retardation of cell growth for the next 48 h (Fig. 2a). In line, the chlorophyll (Chl) content of the culture increased more slowly under −P conditions than under +P conditions (Fig. 2b). The cellular Chl content on an OD_730_·ml basis, therefore, remained lower under −P conditions than under +P conditions throughout the culturing (Fig. 2c). Meanwhile, the total cellular P content, which had initially been 157.1 nmol/(OD_730_·ml), drastically decreased to 24.9 nmol/(OD_730_·ml) in 48 h under −P conditions, relative to a mild reduction to 112.6 nmol/(OD_730_·ml) under +P conditions (Fig. 2d). Despite the severe shortage of P, the survival ratio was little affected in −P cells (95.1%, c.f., 96.4% in +P cells), which demonstrated proper −P-acclimation of *C. kessleri* cells (Fig. 2e).

### P-starvation induced lipid remodeling in *C. kessleri*

**Fig. 3:**
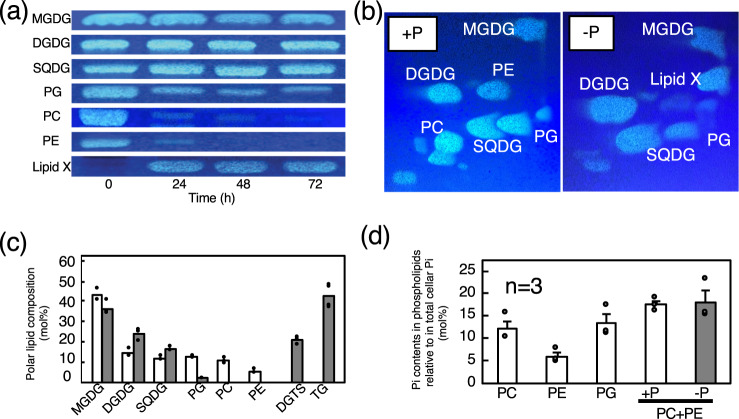
Lipid remodeling in *C. kessleri* cells in response to −P stress. **a** Qualitative TLC analysis of individual polar lipids prepared from cells grown under +P conditions (0 h), and from ones shifted to −P conditions for further growth for 24, 48, and 72 h. Respective lipids came from different regions of the plate (see Supplementary Fig. 2). **b** Two-dimensional TLC profile of polar lipids in the cells grown for 48 h under +P or −P conditions. **c** Polar lipid composition in the cells grown for 48 h under +P (white bars) or −P conditions (gray bars). The content of TG was shown as that of its constituent fatty acids, relative to total polar lipids. **d** The contents of Pi included in individual phospholipids, relative to that in total cellular fraction. The value as to PC + PE under −P conditions indicates the content of Pi derived from the degradation of PC and PE of +P cells, relative to that in total fraction of −P cells. The values shown are averages for two technical replicates for each of two biological replicates (**c**), or averages ± SEM estimated on the basis of the data in Fig. 2d (*n* = 3) and c. Some dots are overlapped (refer to source data in Supplementary Data 1).

**Fig. 4:**
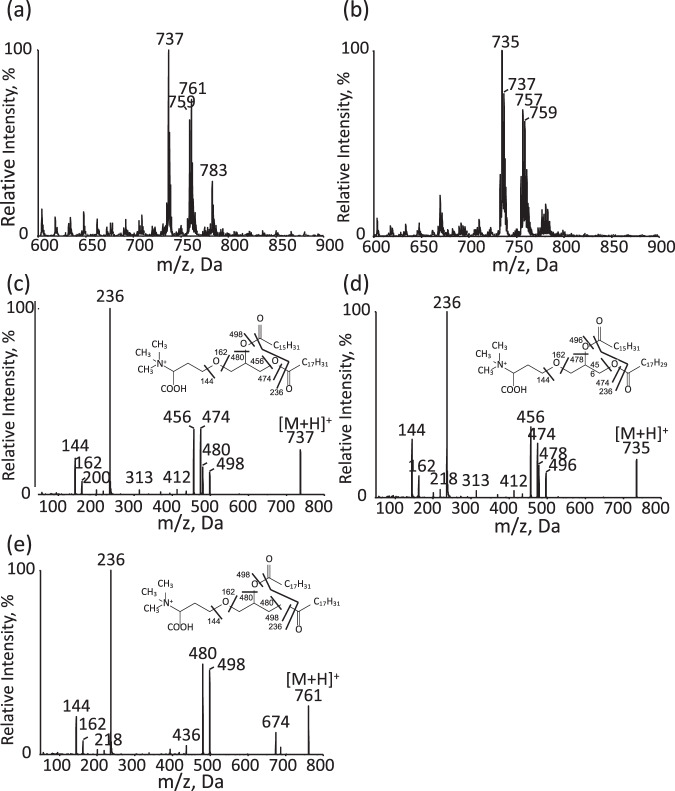
Identification of a −P-induced lipid as DGTS through LC/MS^2^ analysis. ESI mass spectra as to a −P-induced lipid of *C. kessleri* (**a**) and DGTS of *C. reinharditii* (**b**). Product ion spectra (**c**), (**d**), and (**e**), as to *m/z* 737 in (**a**), *m/z* 735 in (**b**), and *m/z* 761 in (**a**), respectively.

We then investigated whether the mechanism by which *C. kessleri* cells acclimate to −P stress involves lipid remodeling. TLC analysis showed that the shift of *C. kessleri* cells from +P to −P conditions brought about the appearance of a lipid at a substantial level at 24 h, followed by persistent maintenance of the level for the next 48 h (Lipid X, Fig. 3a, b, Supplementary Fig. 2). Lipid X, which was novel to the best of our knowledge, was subjected to ESI-MS^2^ analysis. Its ESI mass spectra showed two protonated positive ions, *m/z* 737 and 761, and their respective sodium adduct ions, *m/z* 759 and 783 (Fig. 4a). Meanwhile, DGTS prepared from *C. reinharditii* gave protonated *m/z* 735 and 737 signals and their sodium adducts, *m/z* 757 and 759 (Fig. 4b). Notably, the product ion spectrum at *m/z* 737 for *C. kesssleri* exhibited product ions of m/z 144, 162, and 236, which were common to those of *m/z* 735 in *C. reinhardtii* (Fig. 4c, d). These results allowed us to identify lipid X as DGTS, compatible with a previous report^28^. Accordingly, it was interpreted that the product ions, m/z 456 and 474, and *m/z* 480 and 498, resulted from the loss of 18:2 and 16:0, respectively, in *C. kessleri* (Fig. 4c). Similarly, the product ion spectrum of *m/z* 761 exhibited three signals (*m/z* 144, 162, and 236) leading to its identification as DGTS, and two signals for the loss of 18:2 (*m/z* 480 and 498) (Fig. 4e). These results, together with fatty acid analysis at the *sn*-2 position (see below), clarified that the DGTS molecular species in P-starved *C. kessleri* consisted predominantly of *sn*-1 16:0/*sn*-2 18:2 and *sn*-1 18:2/*sn*-2 18:2 species.

Subsequent quantitative GC analysis of the constituent fatty acids in individual polar lipids showed drastically different lipid compositions between +P and −P cells: PC and phosphatidylethanolamine (PE) amounted to 11.3 and 5.6 mol%, respectively, relative to total polar lipids in +P cells with no DGTS, whereas DGTS accumulated to 21.0 mol% in −P cells with the disappearance of PC and PE (Fig. 3c). Another phospholipid, PG, amounted to 12.6 mol% in +P cells; however, it decreased to only 2.3 mol% in −P cells. In addition, −P cells showed a lower content of monogalactosyl diacylglycerol (MGDG) (36.2 mol%, c.f., 43.6 mol% in +P cells) with higher contents of DGDG (24.0 mol%, c.f., 14.9 mol% in +P cells) and SQDG (16.5 mol%, c.f., 12.0 mol% in +P cells). It thus turned out that *C. kessleri*, as well as other lower green plants, can synthesize DGTS, and that this ability is evident under −P conditions, but not under +P conditions. In addition, typical lipid remodeling at chloroplast membranes occurred in *C. kessleri*, as reported in other photosynthetic organisms, such that PG decreased with a concomitant increase in SQDG, probably to keep the charge of the membranes at a certain level (Fig. 3a–c)^2^. In addition to the above changes in the composition of membrane lipids, the accumulation of triacylglycerol at 42.7% relative to the total content of membrane lipids was characteristic of −P cells (Fig. 3c), in contrast to +P cells where this storage lipid was almost completely absent.

Based on the Pi content of the total cellular fraction and those of individual phospholipids in +P cells, the quantitative proportions of phosphate contents in individual phospholipids to the total cellular fraction were estimated to reach 12.1, 6.0, and 13.5% Pi in PC, PE, and PG, respectively (Fig. 3d). Therefore, PC and PE totally accounted for 18.1% and 17.6% of total Pi in +P cells and −P cells, respectively (Fig. 3d).

### −P-induced DGTS synthesis via eukaryotic pathway

**Fig. 5:**
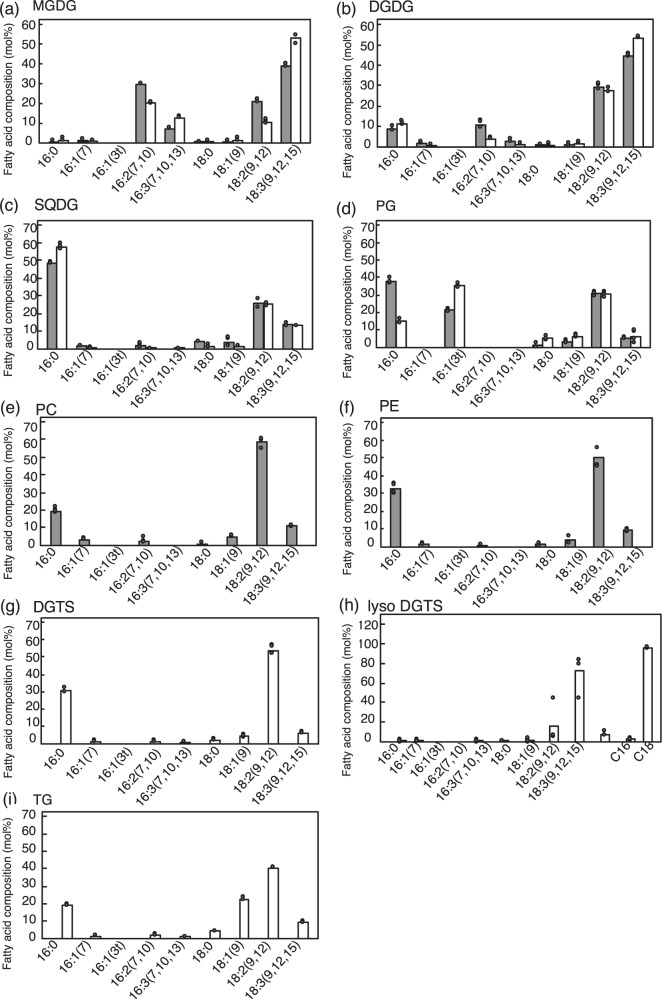
Fatty acid composition of individual polar lipids. The fatty acid compositions of MGDG (**a**), DGDG (**b**), SQDG (**c**), and PG (**d**) are shown for 48-h grown +P (gray bars) and −P cells (white bars), whereas those of PC (**e**) and PE (**f**), and DGTS (**g**) are for +P and −P cells, respectively. The fatty acid composition of *sn*-2 monoacyl lysoDGTS is shown in (**h**). Fatty acid composition of TG in −P cells is shown in (**i**). The values shown are averages for two technical replicates for each of two biological replicates. Some dots are overlapped (refer to source data in Supplementary Data 1).

Individual lipids in +P cells showed their characteristic fatty acid compositions, as previously reported (Fig. 5a–f)^17^. Concerning chloroplast glycolipids, MGDG, in particular, and DGDG contained substantial amounts of unsaturated C_16_ and/or C_18_ acids such as 16:3 and 18:3 (Fig. 5a, b). SQDG had 16:0 and 18:2 as major fatty acids while PG mainly included a sole trans-unsaturated fatty acid, 16:1(3*t*), in addition to 16:0 and 18:2 (Fig. 5c, d). Regarding extrachloroplast lipids, PC and PE mainly contained 16:0 and 18:2 (Fig. 5e, f). Meanwhile, MGDG in −P cells, compared with that in +P cells, contained 18:3 and 16:3 more abundantly at the expense of 18:2 and 16:2, with DGDG and SQDG, respectively, displaying only small effects of −P, if any, on their fatty acid compositions. PG demonstrated a higher content of 16:1(3t) in −P cells than in +P cells at the expense of 16:0. Intriguingly, the fatty acid composition in DGTS in −P cells was almost the same as those in PC and PE in +P cells, such that 16:0 and 18:2 amounted to 20–30 and 55 mol%, respectively (Fig. 5e–g). Meanwhile, triacylglycerol showed C_18_ acids as the main constituent fatty acids in −P cells (Fig. 5i), as well as in *C. kessleri* cells exposed to other ambient stresses^22–24^.

Membrane lipids are synthesized through the prokaryotic and eukaryotic pathways in *C. kessleri* as well as in *A. thaliana*^17,26^. The prokaryotic and eukaryotic pathways are responsible for the synthesis of polar glycerolipids with C_16_ and C_18_ acids esterified, respectively, at the *sn*-2 position of the glycerol backbone. DGTS was then analyzed to determine the fatty acid composition at the *sn*-2 position to estimate the proportions of prokaryotic and eukaryotic lipids (Fig. 5h). The results indicated that the *sn*-2 position was occupied almost exclusively by C_18_ acids and strongly implied that DGTS, similar to PC and PE, was synthesized almost exclusively through the eukaryotic pathway.

### The metabolic mechanism of −P-induced DGTS accumulation in *C. kessleri*

**Fig. 6:**
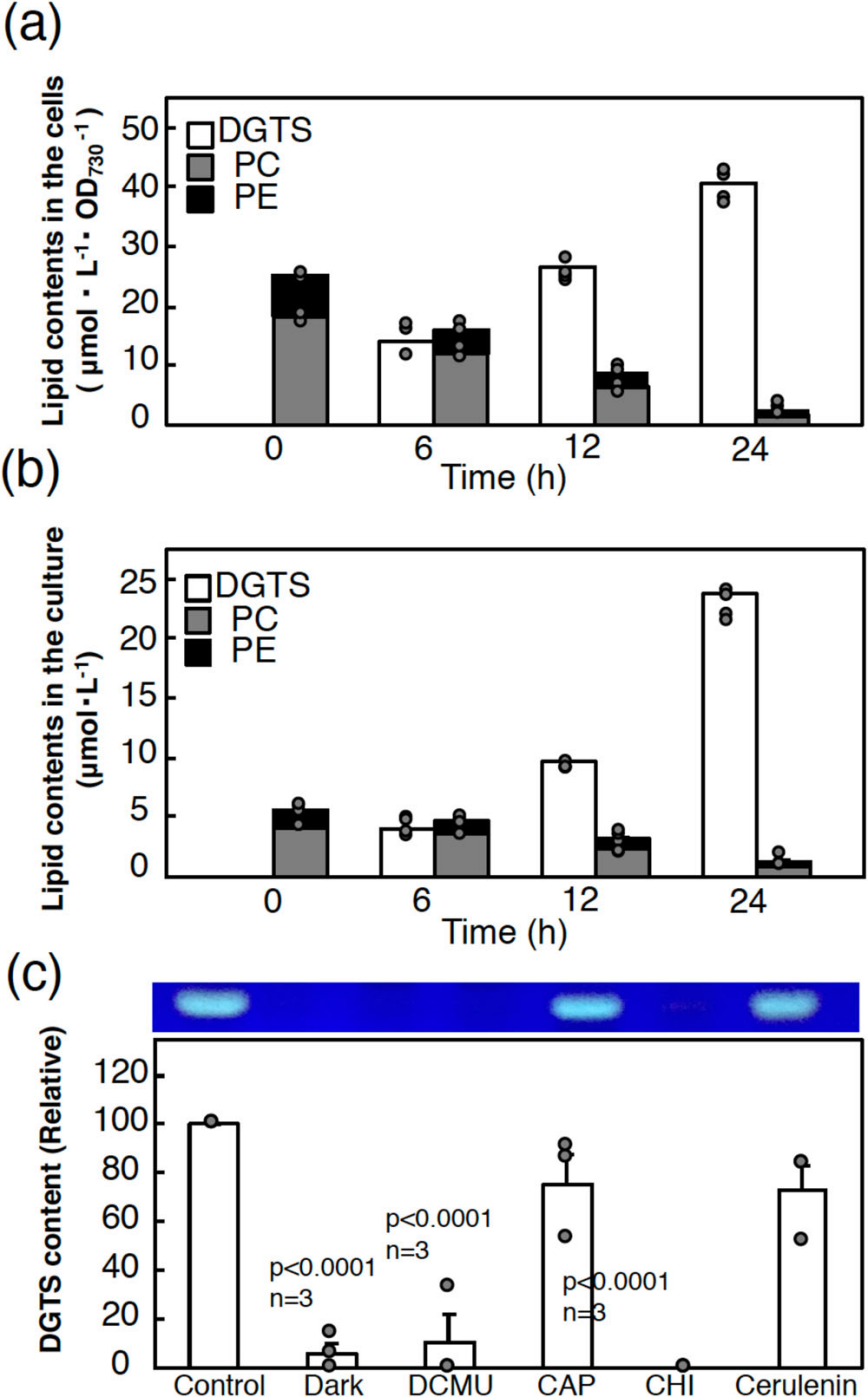
Metabolic mechanism of DGTS accumulation under −P conditions. Changes in the contents of DGTS (white), PC (gray), and PE (black) in cells grown for 24 h after a shift from +P to −P conditions, which were estimated in cells on an OD_730_ L culture basis (**a**), and in the culture (**b**). **c** Effects of metabolic inhibitors or light conditions on the accumulation of DGTS in the cells at 24 h after the shift to −P conditions. The values were estimated as the proportion of the DGTS content in the cells with application of chloramphenicol (CAP), cycloheximide (CHI), cerulenin, and DCMU, and in those shifted to the dark conditions, relative to that in non-treated control cells. The values shown are averages for two technical replicates for each of two biological replicates (**a**, **b**), or averages ± SEM for three biological replicates (**c**). Some dots are overlapped (refer to source data in Supplementary Data 1). Data in (**c**) were analyzed by one-way ANOVA with multiple comparison by Tukey–Kramer’s test.

The structural similarity of the DG moieties of DGTS, and PC and PE raised the possibility that PC and PE release their fatty acid and/or DG moieties upon their degradation for DGTS synthesis. Quantitative changes in these three zwitterionic lipids were then chased for a shorter time within 24 h after the shift of +P cells to −P conditions (Fig. 6a, b). The cellular content of DGTS sharply increased from an initial level of zero at 24 h of −P stress, whereas those of both PC and PE were concomitantly decreased to less than 10% of the initial levels at 24 h (Fig. 6a). Notably, the accumulated content of DGTS was close to the total content of PC and PE decreases, which was compatible with the results in Fig. 3c (48 h). However, the increased level of DGTS at 24 h was more than ca. 5-fold higher than the sum of the initial levels of PC and PE when estimated per ml of culture (Fig. 6b). These results raised the possibility that fatty acids or DG moieties for DGTS synthesis were supplied partially by preexisting PC and PE through their degradation and more abundantly by other metabolic sources, such as de novo fatty acid synthesis.

The effects of metabolic inhibitors on −P-induced DGTS accumulation were investigated to gain insight into the metabolic mechanism of lipid remodeling (Fig. 6c, Supplementary Fig. 3). Cycloheximide, an inhibitor of 80S ribosomes, almost completely repressed DGTS accumulation under −P conditions, whereas chloramphenicol, an inhibitor of 70S ribosomes in chloroplasts or mitochondria, caused only low repression of DGTS accumulation by 24.1%. These results implied that −P-induced DGTS accumulation depended almost completely on the synthesis of nuclear genome-encoded proteins and not so greatly on that of chloroplast or mitochondria genome-encoded proteins. Intriguingly, cerulenin, an inhibitor of β-ketoacyl-ACP synthase of fatty acid synthase, inhibited DGTS accumulation by 27.3%, which inferred involvement not only of de novo synthesized fatty acids but also of fatty acids originating from pre-existing lipids, in the DGTS accumulation, as suggested above. Meanwhile, DGTS accumulation was repressed by 89.0% upon application of 3-(3,4-dichlorophenyl)-1,1-dimethylurea (DCMU), an inhibitor of photosynthesis, or through a shift of the cells to dark conditions, which suggested the dependence of DGTS accumulation almost completely on photosynthesis.

### Identification of the gene for DGTS synthesis in *C. kessleri*

**Fig. 7:**
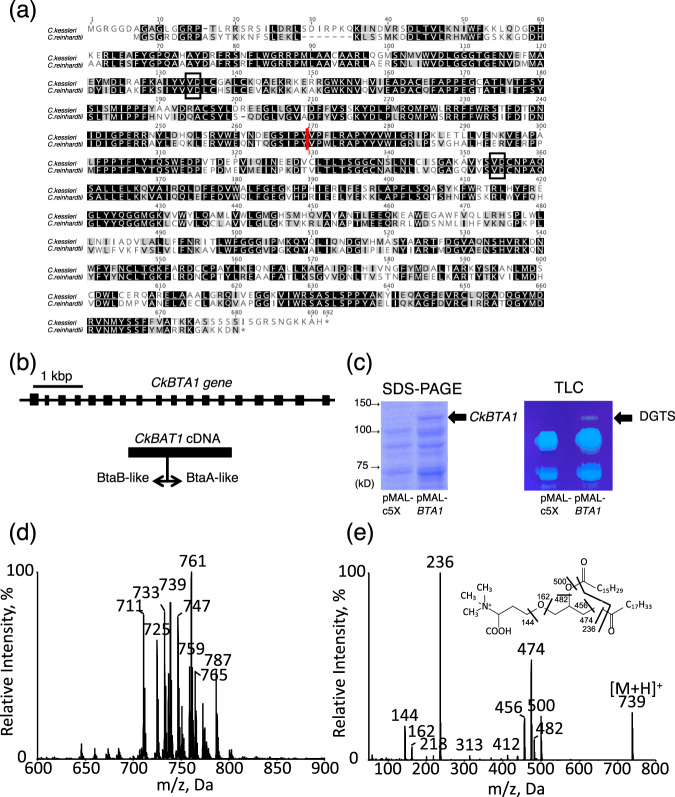
Structural and functional identification of a BTA1 homolog in *C. kessleri*. **a** Alignment of the postulated amino acid sequence of a BTA1 homolog with that of CrBTA1. Identical and similar amino acid residues are shadowed in black and gray. The vertical red line indicates the boundary of the N-terminal BtaB- and C-terminal BtaA-like domains, whereas two black boxes show the conserved dipeptide VD for the binding of SAM, the substrate in the respective catalytic actions of BtaA and BtaB. **b** The structure of the *BTA1* homolog of *C. kessleri*. The *BTA1* homolog is composed of 18 exons on the nuclear genome. **c** Expression of cDNA of the *BTA1* homolog in *E. coli*. SDS-PAGE of total cellular proteins shows induction of the expression of the homolog protein in pMAL-*CkBTA1* introduced *E. coli* cells, but not in empty-vector introduced ones. TLC of total cellular lipids shows the appearance of a lipid specifically in pMAL-*CkBTA1* introduced *E. coli* cells. **d** The ESI mass spectrum of the lipid in the transformant of *E. coli*. **e** The product ion spectrum of *m/z* 739 in (**d**). Note that the product ion spectrum exhibited three signals (*m/z* 144, 162, and 236) for identification of the lipid as DGTS.

We then searched for the gene for DGTS synthesis in the genomic DNA sequence of *C. kessleri* with the use of the protein product of the *C. reinhardtii BTA1* gene (CrBTA1) as a query^29^. We found a DNA region that could encode a protein highly homologous to CrBTA1. cDNA covering this region was synthesized through 5′- and 3′-RACE PCR with the use of total RNA isolated from *C. kessleri* cells starved of P as the template. The primers for 5′- and 3′-RACE PCR were set such that partially overlapping DNA regions (1.5-kbp and 2.0-kbp) were generated. Subsequent determination of the obtained PCR products revealed an ORF, which was postulated to encode a 78.8 kDa protein that comprised 690 amino acid residues (Accession: LC648247). This protein was composed of two domains that were homologous to the bacterial BtaB (S-adenosylmethionine-diacylgycerol homoserine-N-methlytransferase) and BtaA (S-adenosylmethionine-diacylglycerol 3-amino-3-carboxypropyl transferase) proteins at the N- and C-terminal halves, respectively (Fig. 7a). This homolog of *C. kessleri* was thus characterized as type B like *C. reinhardtii* BTA1 (CrBTA1), distinct from type A composed of N-terminal BtaA- and C-terminal BtaB-like domains, and showed 62.1% identity in amino acid sequence with CrBTA1. Furthermore, the consensus sequence VD, for the binding of S-adenosylmethionine as the substrate was conserved in both the BtaA- and BtaB-like domains (Fig. 7a)^29^. The coding region of the full-length cDNA was divided into 18 exons by 19 introns in the genome (Fig. 7b). Owing to these sequence characteristics of this protein product, it was strongly suggested that this homologous gene encodes BTA1 for DGTS synthesis. We then functionally characterized this gene through overexpression of its cDNA in *E. coli* cells that intrinsically lack DGTS. Upon induction of the corresponding protein, a lipid was found to appear in *E. coli* cells (Fig. 7c, Supplementary Fig. 4), which was thereafter identified as DGTS (Fig. 7d, e). These results proved that the homologous gene of *C. kessleri* encodes the BTA1 protein, and thus the gene was designated as *CkBTA1*.

**Fig. 8:**
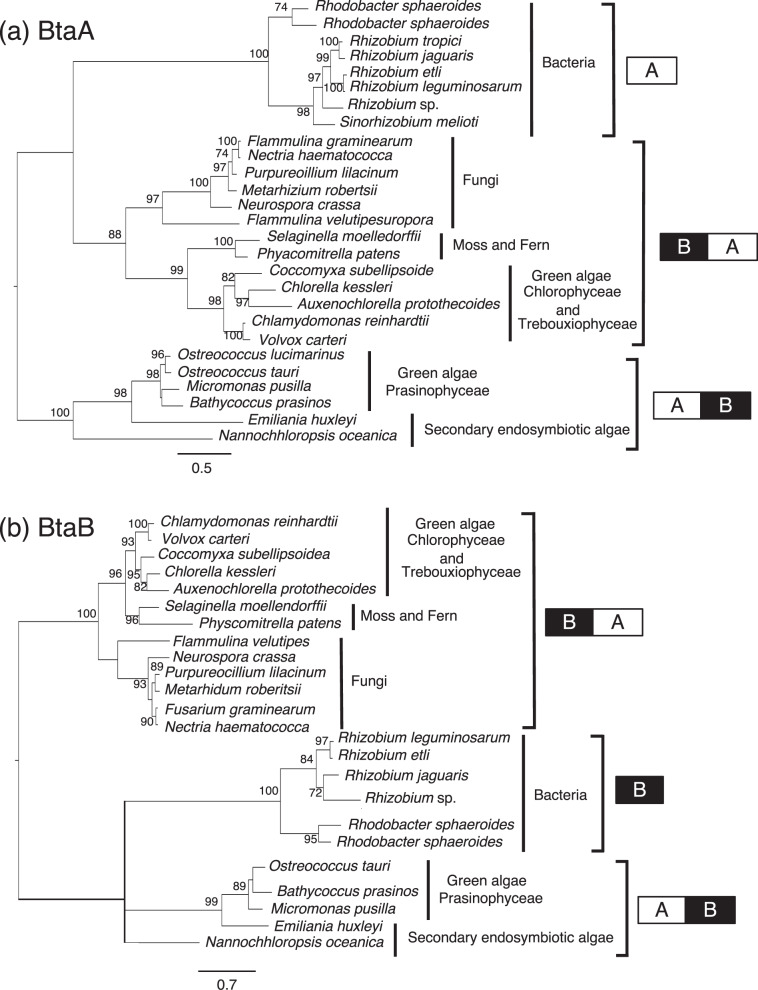
Phylogenetic trees of the BtaA and BtaB domains in BTA1 proteins. **a** Bacterial BtaA and eukaryotic BtaA-domains. **b** Bacterial BtaB and eukaryotic BtaB-domains. Two linked boxes, AB and BA, indicate eukaryotic A and B type BTA1 proteins, respectively, whereas single boxes, A and B, demonstrate bacterial BtaA and BtaB proteins, respectively.

Molecular phylogenetic analysis indicated that the BtaA domain of CkBAT1 is closely related to the counterparts of green algae that belong to Trebouxiophyceae and Chlorophyceae, and to those of nonflowering plants, ferns and mosses (Fig. 8a). However, the clade of green algae including *C. kessleri* was far from the clade of another green algal group, Prasinophyceae, and secondary endosymbiotic algae but rather was close to the clade of fungi. A similar trend was observed for the BtaB domain (Fig. 8b). Meanwhile, bacterial BtaA and BtaB showed single clades.

### Transcriptional upregulation of the genes for −P-induced lipid remodeling

**Fig. 9:**
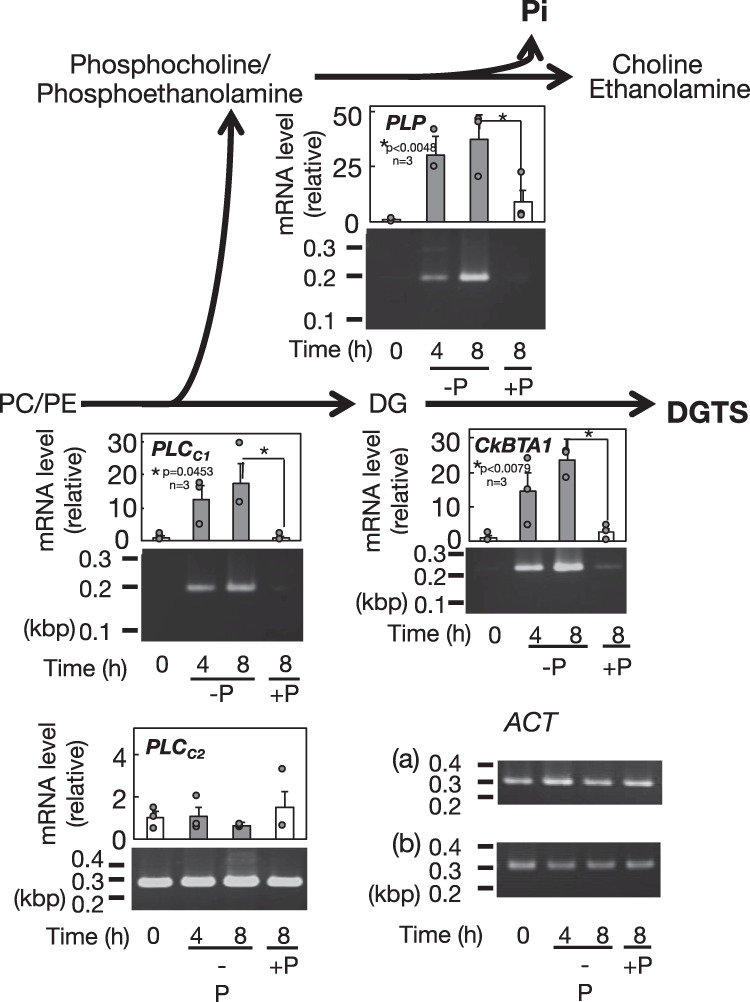
Regulatory expression of the genes for −P induced lipid remodeling in *C. kessleri*. The expression levels of the *BTA1*, *PLC*_*C1*_, *PLC*_*C2*_, and *PLP* genes were investigated through semi-quantitative RT-PCR analysis in *C. kessleri* cells before and after a shift to −P conditions. The intensities of the DNA bands that correspond to mRNAs of the individual genes were used for determination of the values, relative to that of *ACT*. Shown are the values relative to those at 0 h with averages ± SEM for three biological replicates. Some dots are overlapped (refer to source data in Supplementary Data 1). Data were analyzed by one-way ANOVA with multiple comparison by Tukey–Kramer’s test.

Semiquantitative analysis of mRNA levels of individual genes was then performed (Fig. 9, Supplementary Fig. 5). The *CkBTA1* mRNA level was very low under +P conditions, consistent with the complete absence of DGTS, and increased under −P conditions with time, in line with DGTS accumulation. Meanwhile, the degradation of PC and PE might be catalyzed by nonspecific phospholipase C (NPC) and phospholipase D (PLD)^1^. A search for corresponding genes in the *C. kessleri* genome, with *A. thaliana* orthologs of NPC and PLD genes as queries, led us to find that *C. kessleri* has no homologous genes. Alternatively, *C. kessleri* contained two genes coding for proteins homologous to glycerophosphodiester phosphodiesterase (GDPD) with possible PLC activity in *C. reinhardtii*^30^. The expression of one of these candidate *PLC* genes (Cre03.g203600.t1.2, *PLCc*_*1*_), similar to that of *CkBTA1*, was strictly repressed at the transcript level under +P conditions and was upregulated with time after the shift to −P conditions, consistent with the progress of the degradation of PC and PE. Meanwhile, the other candidate gene (Cre16.g683850.t1.3, *PLCc*_*2*_) was little affected at the mRNA level, irrespective of the conditions of P. There was the possibility that phosphocholine and phosphoethanolamine, which might be the degradation products of PC and PE, respectively, via the PLC activity of *PLCc*_*1*_, were subjected to the action of phosphatase for Pi release. In accordance with this idea, the mRNA level of the candidate phosphatase gene *PLP*^31,32^ was markedly upregulated from an almost undetectable level upon −P stress (Fig. 9). These observations implied that the genes responsible for lipid remodeling were subjected to upregulation at least at their transcript levels, which seemed compatible with the dependence of DGTS accumulation on the synthesis of proteins encoded by nuclear genes (Fig. 6c).

Meanwhile, it was previously reported that de novo phospholipid biosynthesis mediated by ER-type lysophosphatidic acid acyltransferase 2 (LPAT2) contributes to lipid remodeling to increase the DGDG content for root growth in *A. thaliana* under −P conditions^33^. The effects of −P on transcript levels were then investigated in *C. kessleri*, as to the genes responsible for the construction of diacylglycerol moieties of phospholipids, i.e., glycerol 3-phosphate acyltransferase (GPAT) and LPAT genes. The expression levels of ER- and plastid-GPAT genes, which are homologous to *A. thaliana GPAT9* and *ATS1*, respectively, were little affected in −P cells relative to in +P cells (Supplementary Fig. 6)^34^. Meanwhile, irrespective of P conditions, the expression levels of ER- and chloroplast-LPAT genes, which corresponded to *CrLPAAT2* and *CrLPAAT1*, respectively, in *C. reinhardtii* were below the detection limit in *C. kessleri* under our experimental conditions^35,36^. These results thus provided no evidence of the regulatory expression of the GPAT or LPAT gene to support de novo phospholipid biosynthesis in *C. kessleri* under −P conditions.

## Discussion

The presence of DGTS has been reported in many lower green plants, with the rare exceptional observation of no detection of DGTS in *C. kessleri* and *C. pyrenoidosa*. However, this study demonstrated that *C. kessleri* cells are able to synthesize DGTS and that this ability is displayed under −P conditions but not under +P conditions (Figs. 3, 4 and 6). This −P-stress-specific DGTS appearance is the reason that DGTS has never been detected thus far in *C. kessleri*^6,16^. It will be necessary to reevaluate the previously reported absence of DGTS in *C. pyrenoidosa*, which might lead to the notion of a general ability for DGTS synthesis in green algae.

In contrast to the prevalent distribution of DGTS in green algae, the presence of DGTS has been reported within only a taxonomically limited range in red algae. In a red microalga, *Galdieria sulphuraria*, DGTS was one of the major polar lipids^37^, which was intriguing with respect to its synthetic pathway since this alga possesses no *BTA1* homolog in its genome (https://phycocosm.jgi.doe.gov/Galsul1/Galsul1.home.html). However, the presence of DGTS has been ruled out in other red microalgae^38–40^. Concerning red macroalgae, the presence of DGTS might have been arguable because of its detection only in limited species and, if any, at very low levels^4,34^. The question of red macroalgal DGTS would be settled with the use of the lipidomic technique^41,42^. Overall, the taxonomic distribution of DGTS should be carefully examined from both biochemical and genetic perspectives, as shown by this study. The use of −P conditions is necessary for biochemical detection of DGTS and its identification in case of strict regulation of the responsible gene, as observed in *C. kessleri*.

As far as lower green plants that possess DGTS are concerned, information on the effects of −P on the DGTS content is limited to that in *C. reinhardti*. Distinct from the secondary endosymbiotic algae and nonphotosynthetic organisms that are known to show −P-induced DGTS increases, *C. reinhardtii* showed little alteration in the DGTS content when shifted to −P conditions, which might imply that this green alga has adapted to −P-stress in freshwater habitats by losing PC^19–21,43^. Therefore, −P-specific induction of DGTS synthesis in *C. kessleri* is the first report among lower green plants. It is necessary to investigate whether a −P-induced increase in DGTS takes place in lower green plants that possess both DGTS and PC under +P conditions (Fig. 1), which would allow us to obtain the whole picture of the lipid-remodeling pattern in lower green plants.

Meanwhile, DGTS was occupied almost exclusively by C_18_ acids at the *sn*-2 position in *C. kessleri* and thus was composed mainly of the eukaryotic type. *C. kessleri* has a homolog of CrLPAAT2 (Supplementary Table 1) with a preference for 16:0 over 18:1 as the acyl group substrate, which, however, would be responsible mainly for the synthesis of triacylglycerol but not for that of membrane lipids including DGTS^36^. Although ER-located LPAAT, which is involved in the synthesis of membrane lipids, has been uncharacterized in green algae, previous radiolabeling experiments and lipid analysis indicated that two lipid biosynthetic pathways, such as those in seed plants, operate in *C. kessleri*^17,26^. Our results would therefore strongly imply that DGTS, similar to PC and PE, was synthesized almost exclusively through the eukaryotic pathway. An in vitro assay demonstrated that DGTS was synthesized not in isolated chloroplasts but in microsomal fractions in *C. reinhardtii*, whereas the counterpart of BTA1 is localized at the ER in a fungus, *F. graminearum*^21,44^. It is, therefore, highly probable that CkBTA1 contributes to DGTS synthesis at extrachloroplast membranes such as ER membranes through the eukaryotic pathway (Fig. 5h). Accordingly, DGTS as well as PC and PE would be present mainly at extrachloroplast membranes in *C. kessleri*, as previously reported in other algal species^10^.

In this context, it was notable that the accumulation of DGTS was accompanied by almost complete degradation of PC and PE and that the relative contents of these two phospholipids, PC and PE, in +P cells were very close to that of DGTS in −P cells (Figs. 3c and 6a). It was previously demonstrated that the viscosity of the lipid phase in DGTS aqueous dispersions was similar to that in PC dispersions through measurement of fluorescence depolarization of parinaric acid^7^. It was thus supposed that DGTS and PC can contribute to the construction of lipid bilayers in biological membranes in a similar manner. In additions, DGTS is similar to PE in terms of its zwitterionic properties. We, therefore, propose that DGTS substitutes for PC, in particular, and PE, as the main bilayer-forming lipid, for the construction of extrachloroplast membranes in *C. kessleri* under −P conditions.

Seed plants adopted another lipid remodeling system at extraplastid membranes in roots and shoots to replace phospholipids such as PC with DGDG^1^. The DGDG content also increased in *C. kessleri*, however, by only 1.6-fold in −P cells relative to +P cells (Fig. 3c; c.f., >8-fold increase of DGDG in roots of *A. thaliana*^45^). This small increase in DGDG might reflect lipid remodeling at chloroplast membranes, in view of a similarly small increase in the DGDG content by 1.2-fold in −P cells of a cyanobacterium, *Synechocystis* sp. PCC 6803^46^. These results thus prompted us to conclude that *C. kessleri*, quite different from seed plants, utilizes DGTS as a main player in the −P-responsive lipid remodeling at extraplastid membranes.

The expression of *PLC*_*C1*_ as the PLC candidate was induced under −P conditions, concomitantly with *PLP* mRNA upregulation. Induced PLC_C1_ and PLP might cooperatively degrade PC and PE to release DG and Pi (Figs. 3 and 9). In line with possible DG release, the chemical structure of DG moieties in terms of the fatty acids of PC and PE was similar to that of DGTS. Therefore, these observations, together with the partial induction of DGTS synthesis with *de novo* synthesis of fatty acids being inhibited (Fig. 6c), prompted us to propose the involvement of PLC_C1_ in DGTS synthesis through release of DG (Fig. 6b). The upregulation of *PLC*_*C1*_ expression was reminiscent of the role of PLC in DG supply through PC degradation in the synthesis of DGTS and DGDG in a bacterium, *Sinorhizobium meliloti*, and *A. thaliana*, respectively, under −P conditions^47,48^. Alternatively, there remains the possibility that the protein designated here as PLC_C1_ is not PLC but has GDPD activity. The GDPD-mediated pathway for phospholipid degradation might function in *C. kessleri*, as previously proposed in seed plants^1,49,50^. Identification of the actual enzymatic activity of PLC_C1_ awaits future study. Meanwhile, −P-induced DGTS synthesis, which would require *de novo* fatty acid synthesis (Fig. 6), might be supported through upregulation of the mRNA level of ER-located acyltransferases, including LPAT, in *de novo* phospholipid biosynthetic pathway^33^. However, no positive evidence was obtained to show −P-induced regulatory expression of *LPAAT2* or *GPAT9* homologs in *C. kessleri*. It was proposed that in *C. reinhardtii*, LPAAT2, which mainly acylates C_16_ acids at the *sn*-2 position of lysophosphatidate, is responsible for the synthesis of TG but not for that of membrane lipids predominantly containing C_18_ acids at their *sn*-2 positions^36^. *C. kessleri* showed −P-induced triacylglycerol accumulation, as is often the case with algal species^51^. The presence of C_16_ acids in −P-induced triacylglycerol (Fig. 5i) might reflect the functionality of LPAAT2 in triacylglycerol synthesis, in view of our previous observation that *C. kesssleri* indeed accumulated triacylglycerol with C_16_ acids at the *sn*-2 position under hyperosmotic and/or nutrient-limitation conditions^24^. Investigation of whether ER-type LPAT for membrane lipid synthesis contributes to −P-induced DGTS synthesis will await identification of another ER-type LPAT that is involved in membrane lipid synthesis in *C. kessleri*.

In view of the Pi content included in PC and PE (18.1% of total cellular Pi), the involvement of PLP in their degradation would enable *C. kessleri* cells to enlarge a Pi pool under −P conditions, thereby giving a great advantage for −P acclimation. Major phospholipids, i.e., PC, PE, and PG, accounted for 31.6% of the total cellular Pi in *C. kessleri*, which was similar to the counterparts occupying 23% of the total Pi in the photosynthetic tissues in seed plants (Fig. 3d)^52^. In addition to phospholipids, nucleic acids occupy 35% of the total Pi in the photosynthetic tissues of seed plants, with more than 85% attributed to rRNA. Accordingly, the major RNases RNS1 and RNS2 played important roles in rRNA degradation for the remobilization of Pi^53^. It will be necessary in the future to investigate Pi-scavenging systems other than DGTS-lipid remodeling in *C. kessleri* as a model green alga, which would lead to a comprehensive understanding of Pi-sequestering systems that is necessary for obtaining the whole picture of the −P-acclimation mechanism in lower green plants.

The convincing proof of DGTS occurrence in lower green plants, together with increasing information on their possession of *BTA1* homologs, prompted us to propose that DGTS synthesis ability appeared in green algae during the evolution of a green lineage. However, the origin of *BTA1* seems different between Chlorophyceae/Treboxiophyceae and Prasinophyceae, as judged from the respective molecular phylogenetic characteristics of the BtaA and BtaB domains, and the fusion order of these two domains in BTA1 proteins (Fig. 8). From the phylogenetic trees of the BtaA and BtaB domains in BTA1, it might be interpreted that, through the evolution of a green lineage, type B BTA1 was first acquired by some ancestral green alga and thereafter inherited by the Chlorophyceae and Treboxiophyceae and finally ferns and mosses, however, without further inheritance by seed plants.

Regarding the composition of zwitterionic lipids, DGTS and PC, lower green plants possessing type B BTA1 can be grouped into three groups, i.e., those containing DGTS only, both DGTS and PC, and PC only, respectively, under +P conditions. One idea is that the first species originally containing PC acquired the *BTA1* gene and then evolved to persistently possess both DGTS and PC, as in, e.g., *D. salina*. Later, it seems that green algae selected one of these two zwitterionic lipids for membrane construction under +P conditions: some species, such as *C. reinhardtii*, have evolved to contain DGTS only by abandoning PC synthesis with the loss of the PC synthesis genes^43^, while other species, such as *C. kessleri*, have chosen PC by repressing the expression of *BTA1*.

It is possible that some ancestral lower green plants, such as *C. kessleri*, lost the *BTA1* gene to evolve into extant seed plants, with the acquisition of the DGDG-utilizing lipid remodeling system. The acquisition of this new remodeling system would have had a beneficial effect on nitrogen (N)-economization and thus on acclimation to N-limitation stress generally encompassing plant habitats, since DGDG, unlike DGTS, includes no N atom. Simultaneously, complete replacement of DGTS with PC, along with the selection of DGDG-utilizing lipid remodeling, might have merited the evolutionary appearance of extant seed plants, since a variety of their biological processes, such as stress acclimation and plant development, are mediated through PC-related signaling pathways with the actions of phospholipases^54^.

## Methods

### Strain and growth conditions

*C. kessleri* 11 h was photoautotrophically grown with 4-fold diluted Gamborg’s B5 (GB5, +P) medium or −P medium made through replacement of NaH_2_PO_4_·2H_2_O with equimolar KCl^23,24^. The OD_730_ value and Chl content were measured to monitor cell growth^24^. The cells were precultured in +P medium to an OD_730_ value of ca. 0.5 and then adjusted to an OD_730_ value of 0.2 with +P or −P medium for further growth. The survival ratio of the cells in culture was examined through 5 μM SYTOX staining (Invitrogen Molecular Probes^55^). When indicated, the culture was supplemented with cycloheximide (8 μg ml^−1^), chloramphenicol (100 μg ml^−1^), cerulenin (10 μM), and DCMU (50 μM) as metabolic inhibitors or shifted to dark conditions simultaneously with the commencement of −P culturing.

### Quantitation of Pi in *C. kessleri* cells

Cells were harvested from a 20 mL culture by centrifugation after washing three times with −P medium and thereafter disrupted with a Beads Crusher µT-12 (Taitec, Saitama, Japan) in 2 mL of extraction buffer comprising 5 mM HEPES‐NaOH, pH 7.5, and 10 mM NaCl^24^. To 100 μL of each whole cell extract, 2 mL of 15 mM potassium peroxodisulfate and 5 mL of distilled H_2_O were added, and the resultant solution was subjected to autoclave treatment (121 °C, 30 min) for the release of Pi from P-containing compounds. To 100 μL of the autoclave-treated solution, 300 μL of a 1.22% malachite green G (Wako, Tokyo) solution and 600 μL of distilled H_2_O were added, and then the resultant solution was left to stand for 30 min. The Pi content was determined in the marachite-green staining solution through spectroscopic measurement of the absorbance at 639 nm^56^.

### Separation and quantitation of individual polar lipids

Cells were harvested at the indicated times for the extraction of total lipids, which were then used for separation of individual polar lipids by two-dimensional TLC^57^. A lipid that appeared under −P conditions was identified through mass spectrometric analyses. Samples were diluted in IPA/MeOH/H_2_O (5:4:1, v/v/v) containing 10 mM ammonium acetate and then directly infused at 10 µL/min flow into a triple quadrupole linear ion trap mass spectrometer (MS) equipped with an electrospray ionization (ESI) source (3200Q with a Trap Turbo V ion source; Sciex, CA). The optimized parameters for DGTS under positive and negative ionization conditions were as follows: ion spray voltage, 5500 V (positive) and −4500 V (negative); declustering voltage, 100 V (positive) and −55 V (negative); and temperature, ambient (both positive and negative). The collision energies for product ion scanning were 60 V (positive) and −45 V (negative). The mass range was scanned *m/z* 600–900 in enhanced mass scan (EMS) mode for precursor ions and *m/z* 50–900 in enhanced product ion scan (EPI) mode for product ions. The mass spectrum data were analyzed with reference to DGTS of *Chlamydomonas reinhardtii* 137c and that of chlorarachniophytes^28^. DGTS from *C. kessleri* was used for the preparation of *sn*-2 monoacyl lysoDGTS through TLC after treatment with *Rhizomucor miehei* lipase (Sigma-Aldrich)^17^. Fatty acid methyl esters derived from total lipids, individual polar lipids or *sn*-2 monoacyl lysoDGTS were quantified by capillary GLC based on their constituent fatty acids^17^.

### Cloning of cDNA for BTA1, determination of its nucleotide sequence, and its expression in *E. coli*

A BLAST search was performed with the amino acid sequence of BTA1 of *C. reinhardtii* (CrBTA1) as a query in the genomic DNA database of *C. kessleri* (https://blast.ncbi.nlm.nih.gov/Blast.cgi?PROGRAM=tblastn&PAGE_TYPE=BlastSearch&BLAST_SPEC=Assembly&LINK_LOC=blasttab&ASSEMBLY_NAME=GCA_001598975.1). Sixteen regions that aligned closely on the genome were hit and therefore, as a whole, were postulated to encode a BTA1 homolog. One of these regions was chosen for primer setting for 5′- and 3′-race PCR with a SMARTer RACE 5′/3′ kit (Takara, Tokyo; primer set 1, Supplementary Table 1), and the nucleotide sequence of the amplified DNA was determined with a BigDye Terminator v3.1 cycle sequencing kit on a 3500 genetic analyzer (Thermo Fisher, Tokyo).

For heterologous expression of *BTA1* in *E. coli*, the ORF of the *BTA1* cDNA was amplified by PCR with primer set 2 (Supplementary Table 1) and then ligated to a pMAL-c5X vector (New England Biolabs Japan, Tokyo) to generate the vector designated as pMAL-*BTA1*. The pMAL-*BTA1* or empty pMAL-c5X vector was introduced into NEB express competent *E. coli* cells (New England Biolabs Japan, Tokyo). Transformed cells were subjected to the induction of gene expression with IPTG, and then to SDS-PAGE analysis of total cellular proteins, as described in the manufacturer’s manual, and to TLC analysis of lipids.

### Phylogenetic tree

For phylogenetic analysis, the amino acid sequences of BTA1 homologs were searched in available databases with CrBTA1 (CHLREDRAFT_77062) and BtaA (RSP_0856) and BtaB (RSP_0857) of *Rhodobacter sphaeroides* 2.4.1 as queries. The homolog sequences of CrBTA1 obtained are summarized in Supplementary Table 2. The sequences were aligned after editing, including the deletion of regions with low conservation among these sequences, with Geneious 9.1.8 (Tomy Digital Biology, Tokyo), and thereafter, the aligned sequences were subjected to phylogenetic analysis with IQ-tree 1.6.12 by the maximum-likelihood method^58^.

### Semiquantitative PCR analysis

Total RNA was extracted from *C. kessleri* cells by phenol–chloroform extraction for cDNA synthesis by random primer-based reverse-transcription^24^. Subsequently, the synthesized cDNA was used as a template for semiquantitative RT-PCR. Specific forward (F) and reverse (R) primer sets 3–6 were designed for the respective genes (Supplementary Table 1) on the basis of the information on the above-described genomic DNA sequences of *C. kessleri*. The amplified DNA fragments were separated by agarose gel electrophoresis and then subjected to staining with ethidium bromide to obtain a fluorescent image by photography^59^. The fluorescence intensities of DNA bands for the individual genes were estimated with ImageJ (http://rsbweb.nih.gov/ij/) relative to that for the β-actin gene (*ACT*) as an internal control.

### Statistics and reproducibility

The number of biological replicates were 3 to 4 for statistical analysis, with sample sizes being 3–4, as described in figure legends. Data were analyzed by one-way ANOVA, which was followed by multiple comparison by Tukey–Kramer’s test. The data when statistically analyzed are reported as the mean ± SEM.

### Reporting summary

Further information on research design is available in the Nature Research Reporting Summary linked to this article.

## Supplementary information

Supplementary Information

Description of Additional Supplementary Files

Supplementary Data 1

Reporting summary

## Data Availability

Source data of figures including Supplementary Fig. 3 are presented in Supplementary Data 1. The nucleotide sequence of *CkBTA1* cDNA with the postulated amino acid one of its product was deposited in GenBank (LC648247)^60^.
