# Supplementary material for: Diacylglyceryl-N,N,N-trimethylhomoserine-dependent lipid remodeling in a green alga, Chlorella kessleri
Source: Commun Biol. 2022 Jan 11;5:19. doi: 10.1038/s42003-021-02927-z (PMC8752610; doi:10.1038/s42003-021-02927-z)
Supplement: Supplementary file 1 — Supplementary Information [file 42003_2021_2927_MOESM1_ESM.pdf]

Supplementary Table 1 Primer set

| set | gene | Forward | Reverse | Query |
|---|---|---|---|---|
| 1 | *5′ race cDNA* | ctaatacgactcactatagggcaagcagtggtatcaacgcagag | gattacgccaagcttcagtcactgggtcctcccacgact | |
| 2 | *3′ race cDNA* | gattacgccaagctttcttcccgcccaccttcctgtac | ctaatacgactcactatagggcaagcagtggtatcaacgcagag | |
| 3 | *CkBTA1* | gcgcagactaattcgatgggacgcggtggagat | tgttgttgttgttcgtcagtgggccttcttgcc | |
| 4 | *BTA1* | atgtgattgcatccccatc | ccaatgtcgatgttgtctgtg | Cre.07.g324200.t1.2 |
| 5 | *PLCC1* | gctctcatggtagccgactc | tttagctccttcgaccctga | Cre03.g203600.t1.2 |
| 6 | *PLCC2* | catcttccctgaccacac | ccgttgaatgcctggttg | Cre16.g683850.t1.3 |
| 7 | *PLP* | cacccctaccatgagtgcag | gccagcacaacgtcttgc | AT1G17710 |
| 8 | *ACT* | agaaggacacttacattggc | cagagtcaagcacaataccg | AB046457.1 |

Supplementary Table 2 BtaA, BtaB, and BTA1 homologs

| | Organisms | gene ID |
|---|---|---|
| BtaA | **Bacteria** | |
| | *Rhodobacter sphaeroides ATCC 17029* | *Rsph17029_2516* |
| | *Rhodobacter sphaeroides ATCC 17025* | *Rsph17025_3616* |
| | *Rhizobium* sp S41 | BA939_05055 |
| | *Rhizobium leguminosarum bv. trifolii* WSM2304 | Rleg2_2613 |
| | *Rhizobium jaguaris* | CCGE525_12990 |
| | *Rhizobium tropici* | RTCIAT899_CH11455 |
| | *Rhizobium etli bv. mimosae Mim1* | REMIM1_CH02915 |
| | *Sinorhizobium meliloti 2011* | NP_386300 |
| BtaB | **Bacteria** | |
| | *Rhodobacter sphaeroides ATCC 17029* | Rsph17029_2515 |
| | *Rhodobacter sphaeroides ATCC 17025* | Rsph17025_3617 |
| | *Rhizobium-sp NT-26* | NT26_2169 |
| | *Rhizobium leguminosarum bv. trifolii WSM2304* | *Rleg2_2614* |
| | *Rhizobium jaguaris* | CCGE525_12995 |
| | *Rhizobium etli by phaseoli* | IE4803_CH03093 |
| BTA1 | **Green algae** | |
| | *Chlamydomonas reinhardtii* | CHLREDRAFT_77062 |
| | *Coccomyxa subellipsoidea* | COCSUDRAFT_28345 |
| | *Auxenochlorella protothecoides* | F751_5248 |
| | *Volvox carteri f. nagariensis* | VOLCADRAFT_83033 |
| | *Ostreococcus lucimarinus* | OSTLU_27669 |
| | *Ostreococcus tauri* | OT_ostta15g02860 |
| | *Micromonas pusilla* | MICPUCDRAFT_48920 |
| | *Bathycoccus prasinos* | Bathy08g00260 |
| | *Monoraphidium neglectum* | MNEG_12083 |
| | **Fern** | |
| | *Selaginella moellendorffii* | SELMODRAFT_429378 |
| | **Moss** | |
| | *Physcomitrella patens subsp. Patens* | 112274025 |
| | **Secondary endosymbiotic algae** | |
| | *Emiliania huxleyi* | EMIHUDRAFT_105120 |
| | *Nannochloropsis oceanica* | LC375792 |
| | **Fungi** | |
| | *Metarhizium robertsii* | MAA_07461 |
| | *Purpureocillium lilacinum* | VFPFJ_09846 |
| | *Colletotrichum fioriniae* | CFIO01_08357 |
| | *Nectria haematococca* | NECHADRAFT_31251 |
| | *Fusarium graminearum* | FGSG_00742 |
| | *Flammulina velutipes* | KM668875 |
| | *Neurospora crassa* | NCU03032 |

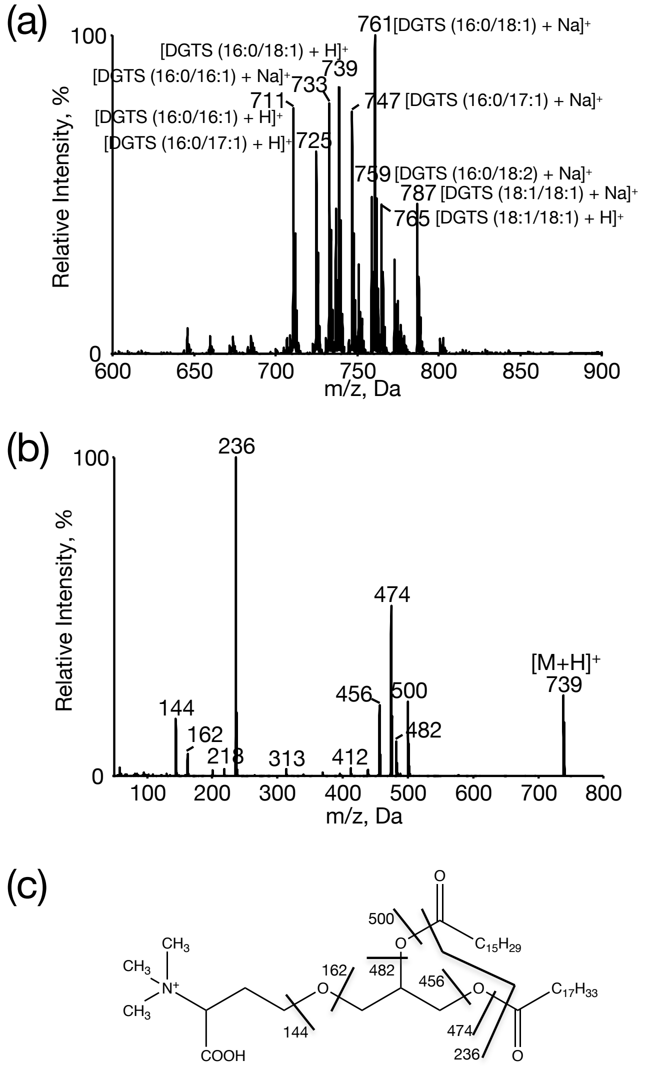


Supplementary Figure 1 Mass spectrometric identification of DGTS. (a) ESI mass spectra of DGTS expressed in *E. coli*. (b) Product ion spectrum of m/z 739 at (a). (c) Estimated fragmentation pattern for DGTS (16:0/18:1) from the product ion spectrum, in which the *sn* position of fatty acids is provisional.

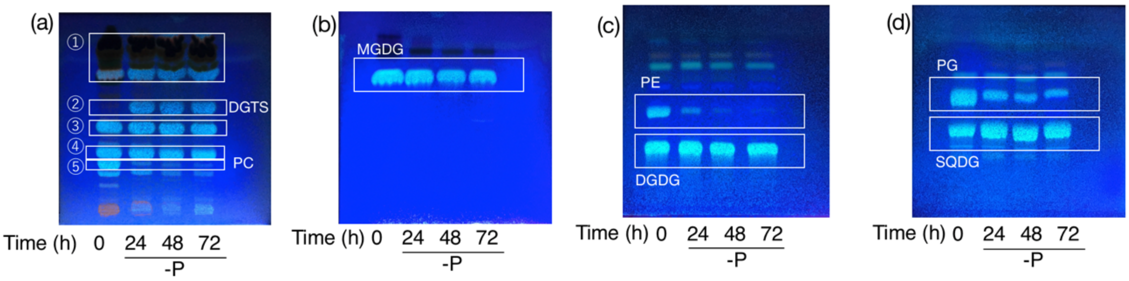


Supplementary Figure 2  Effects of -P on respective lipid contents in *C. kessleri*. Total lipids were extracted from cells before the shift to -P conditions (0 h) or at indicated times after the shift (24-72 h). (a) Total lipids were then separated into DGTS (Band 2) and PC (Bands 5) and also into mixed lipid classes (Bands 1, 3, and 4) through TLC with the solvent system of $CH_3Cl/CH_3OH/H_2O$ (65:25:4, by vol.). Mixed lipids extracted from silica gels of Bands 1, 3, and 4 were respectively subjected to TLC analysis with the solvent system of $CH_3Cl/CH_3OH$/conc. $NH_3$ solution (65:35:5, by vol.). Bands 1, 3, and 4 included MGDG (b), PE and DGDG (c), and PG and SQDG (d), respectively.

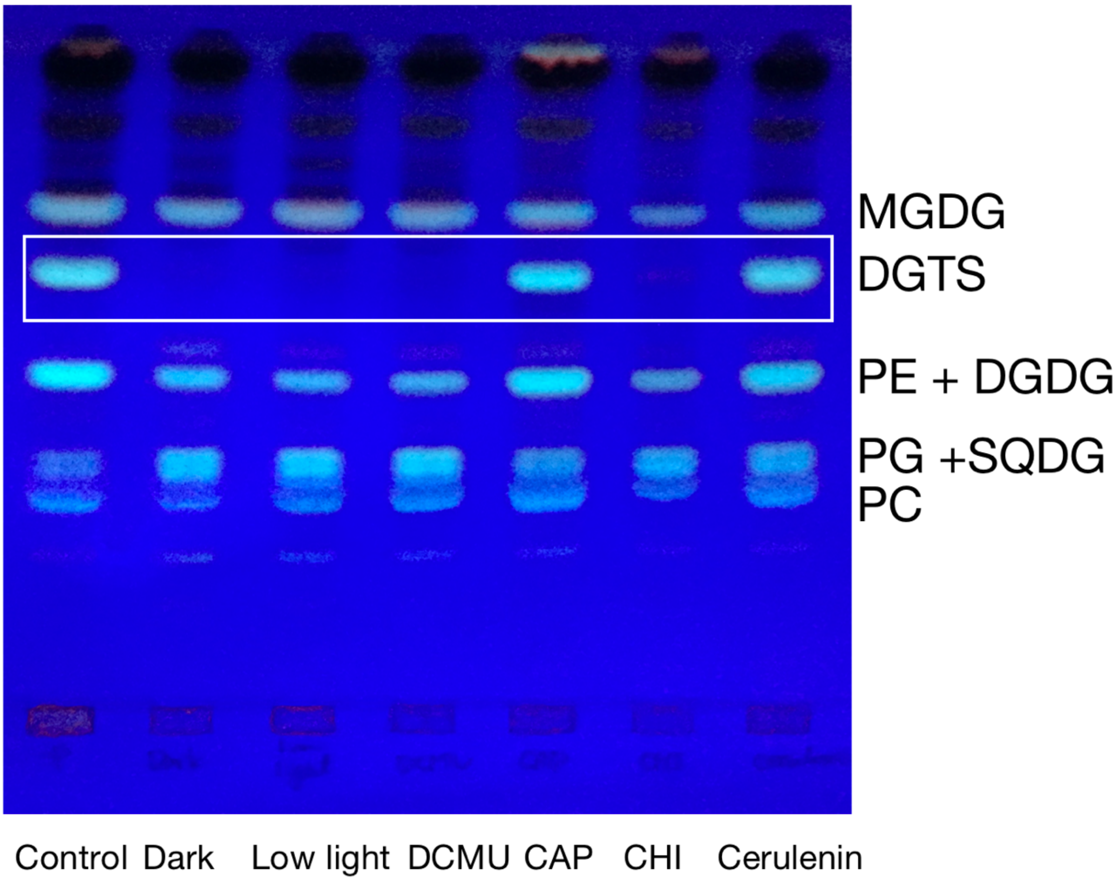


Supplementary Figure 3 TLC profile of polar lipids in *C. kessleri* cells at 24 h after the shift to -P conditions. The cells were cultured under normal conditions (Control), in the dark or low light conditions, or with application of DCMU, chroramphenicol (CAP), cycloheximide (CHI) or celurenin.

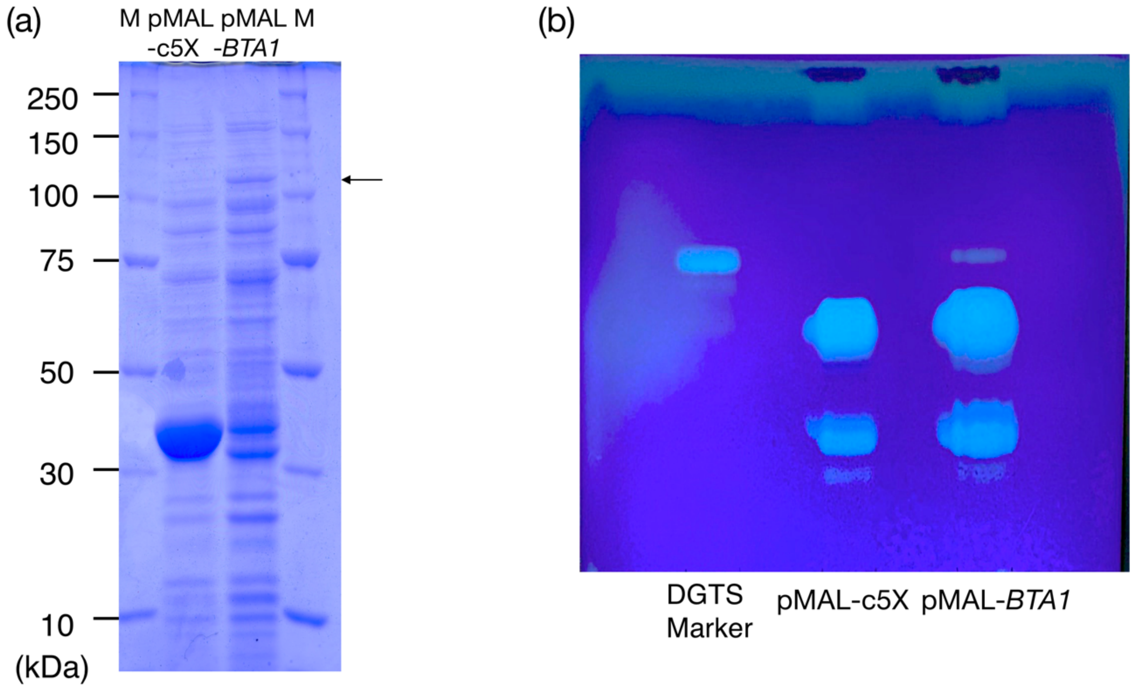


Supplementary Figure 4 Characterization of the *BTA1* homolog of *C. kessleri* in *E. coli*. (a) SDS-PAGE of total cellular proteins in *E. coli* cells having a pMAL-c5X empty vector or pMAL-*BTA1* vector. The former cells overexpressed maltose-binding protein (ca. 40 kDa) whereas the latter ones overexpressed the BTA1 homolog fused with the maltose-binding protein (see arrow). M indicates size markers. (b) TLC profile of polar lipids in transformed *E. coli* cells.

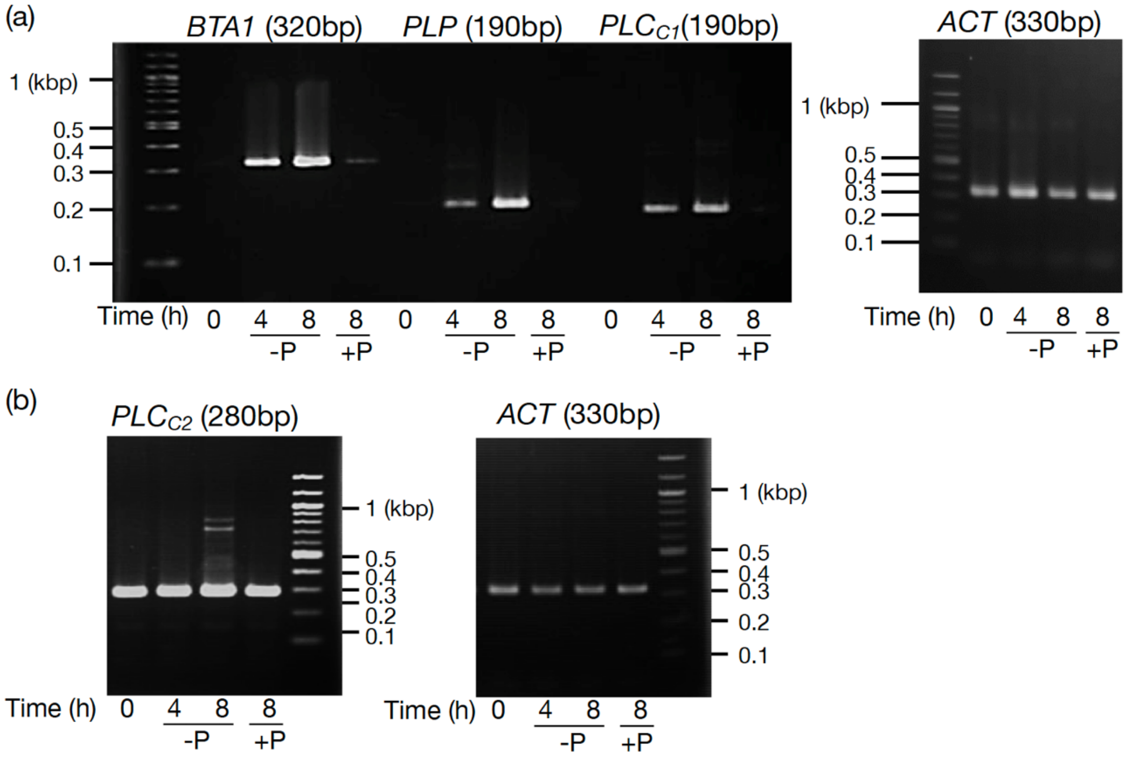


Supplementary Figure 5 Effects of -P on the expression of *BTA1*, *PLP*, and $PLC_{C1}$ genes (a), and that of $PLC_{C2}$ gene (b) in *C. kessleri*. Shown are images of agarose gel electrophoresis of DNA bands that correspond to mRNAs of the individual genes. The intensities of the DNA bands were used for determination of the values, relative to that of *ACT*, to obtain data of Figure 9.

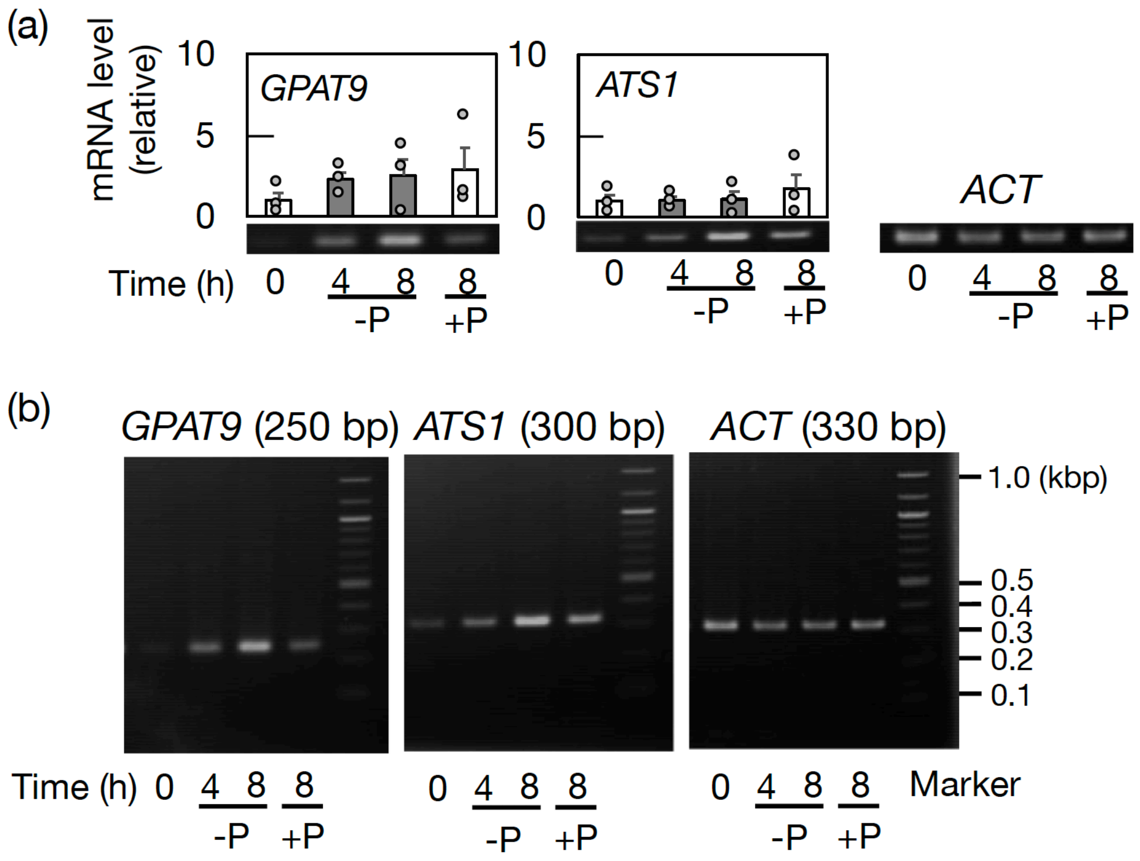


Supplementary Figure 6 Effects of -P on the expression of *GPAT* and *ATS1* genes in *C. kessleri*. (a) The intensities of the DNA bands that correspond to mRNAs of the individual genes were used for determination of the values, relative to that of *ACT*. The values are expressed as averages ± SEM for three biological replicates. (b) Images of agarose gel electrophoresis of DNA bands to obtain data for (a).
