# Supplementary material for: Diacylglyceryl-N,N,N-trimethylhomoserine-dependent lipid remodeling in a green alga, Chlorella kessleri
Source: Commun Biol. 2022 Jan 11;5:19. doi: 10.1038/s42003-021-02927-z (PMC8752610; doi:10.1038/s42003-021-02927-z)
Supplement: Supplementary file 2 — Description of Additional Supplementary Files [file 42003_2021_2927_MOESM2_ESM.pdf]

Description of Additional Supplementary Files

File name: Supplementary Data 1.
Description: Source data for all figures, including supplementary ones.
